# Outcome of Surgical Treatment for Metastatic Bone Disease of the Forearm

**DOI:** 10.1002/cnr2.70208

**Published:** 2025-04-24

**Authors:** Jennifer Sebghati, Panagiotis Tsagkozis

**Affiliations:** ^1^ Karolinska University Hospital Solna Sweden; ^2^ Department of Molecular Medicine and Surgery Karolinska Institute Stockholm Sweden

**Keywords:** bone metastasis, forearm, pathological fracture, surgery

## Abstract

**Background:**

Metastatic bone disease and pathological fractures in the long bones of the forearm are rare. The methods and outcomes of surgical treatment for these fractures have not been adequately described.

**Aims:**

To analyze the outcome of surgery for pathological fractures of the forearm.

**Methods and Results:**

Retrospective study of 30 complete and impending pathological fractures (28 consecutive patients) in the forearm, operated on in a single tertiary center between 1986 and 2020. The most common malignancy was hematological disease (multiple myeloma and lymphoma). Most fractures (*n* = 19) were managed with plate and screw reconstruction. In some cases, simple curettage or segmental resections of the metastasis were performed. Local complications were noted in six operations, the most common one being tumor relapse seen in three patients. Most patients had good outcomes regarding restoration of function and pain relief. There were no secondary surgeries in segmental resection, and the function was near normal.

**Conclusion:**

Surgical reconstruction of metastases in the long bones of the forearm usually results in a good functional outcome with an acceptable complication rate. Plate osteosynthesis is often indicated. Segmental excision can be reserved for dispensable parts of the ulna and radius, with excellent results.

## 1 Introduction

Metastatic bone disease (MBD) can lead to symptoms including local pain and pathological fractures [1, 2]. MBD is more commonly diagnosed in bones composed of a higher percentage of red marrow and trabecular bone [1] such as the axial skeleton, the femur, and the humerus [3]. The occurrence of bone metastases in the distal extremities is rare [4]. While there is some data regarding the lower leg and hand, very little is known regarding lower arm MBD; the incidence has been reported to be 0.01%–2% in the ulna and 0.4%–1% in the radius [5, 6, 7, 8]. Moreover, a study reported the frequency of pathological fractures involving the lower arm to be 2 out of 34 fractures (6%) [9]. Surgical management of MBD in the lower arm is poorly documented in the medical literature and mainly through case reports. We set out to describe our experience in the surgical treatment of MBD of the lower arm to provide a background that may aid clinicians and patients in decision‐making.

## 2 Materials and Methods

### 2.1 Study Design and Data Collection

This retrospective cohort study was based on prospectively collected data in the Bone Metastasis Registry. Complementary information was collected from electronic patient records such as the primary diagnosis, the precise location of the lesion, operation date, surgical method, adjuvant radiotherapy, date of death, potential complications and reoperations, date of reoperation, postoperative pain, and restoration of mobility. Pain was categorized in a 4‐grade scale (none, mild, moderate, and severe) whereas mobility was categorized in three groups (normal, minor deficit, major deficit). The study was conducted according to the ethical permits 2012/272‐31 and 2019‐06189 of the Regional Ethics Committee.

The inclusion criteria were fractures to the radius or ulna caused by bone metastases, which included both complete and impending pathological fractures. Between 1986 and 2020, a total of 28 patients (30 fractures) had surgical treatment at Karolinska University Hospital because of pathological or impending fractures in the lower arm caused by MBD.

### 2.2 Statistical Analysis

Statistical analysis was done in SPSS v.28 (IBM). In the cohort, two patients suffered from multiple fractures. Therefore, the fracture location, surgical method, and function outcomes were statistically based on the total number of fractures (*n* = 30). This was also applied regarding the frequency of reoperations, local complications, and the calculated reoperation rate. Parameters such as age, gender, primary diagnosis, and survival rate were calculated based on the number of included participants (*n* = 28). Survival and reoperation rates were calculated as per Kaplan–Meier, with the use of the log‐rank test for comparison between groups. Confidence intervals (CIs) of 95% were calculated. All tests were double‐sided, and a *p* value of ≤ 0.05 was considered significant.

## 3 Results

### 3.1 Patient Characteristics

Twenty‐eight patients (11 women) were included in the cohort (Table 1). At the time of treatment, the median age was 71 years (range 43–86). The most common diagnosis (10 patients) was hematologic malignancy, which included diseases such as myeloma (six patients), lymphoma (two patients), myeloid sarcoma (one patient), and myeloproliferative disorder (one patient). Other diagnoses were renal cancer (six patients), lung cancer (five patients), breast cancer (two patients), melanoma (two patients), and other malignancies such as prostate cancer, liver cancer, and Merkel‐cell carcinoma (one patient each).

**TABLE 1:** Patient characteristics. Data are presented as the frequency of occurred events and percentages (%).

Patient characteristics		Number (%)
Patients	Total	28
	Female	11 (39%)
	Male	17 (61%)
Age at treatment	Median (range)	71 (43–86)
Primary diagnosis	Hematologic malignancy	10 (36%)
	Renal cancer	6 (21%)
	Lung cancer	5 (18%)
	Breast cancer	2 (7%)
	Melanoma	2 (7%)
	Other primary tumors	3 (11%)
Fractures	Total	30
Location	*Radius*	18 (60%)
	Proximal	6 (20%)
	Diaphysis	6 (20%)
	Distal	3 (10%)
	Unspecified	3 (10%)
	*Ulna*	12 (40%)
	Proximal	4 (13%)
	Diaphysis	4 (13%)
	Distal	2 (7%)
	Unspecified	2 (7%)

In the cohort, two patients with renal cancer suffered from multiple fractures. One of them had fractures in both the ulna and radius in the same arm, while the other one had a fracture in each arm. Out of these 30 fractures, 18 were located in the radius and 12 in the ulna. At the time of the last follow‐up, 1 patient was still alive and 27 were deceased.

### 3.2 Oncologic Outcome

**FIGURE 1:**
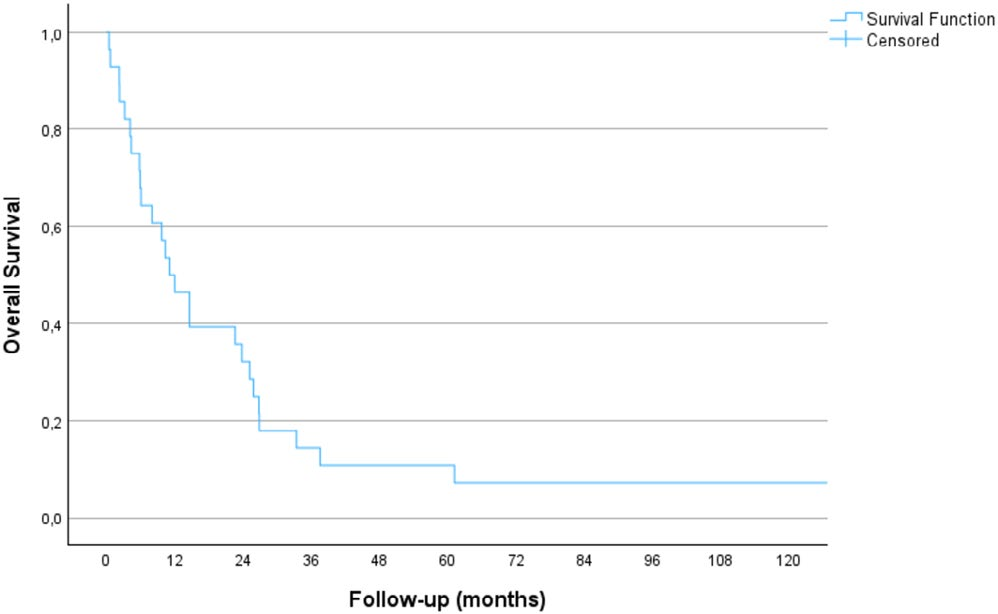
Overall patient survival after primary operation, as per Kaplan–Meier.

The median time between tumor diagnosis and surgery was 57 months (range 1–229). Median follow‐up time was 11 months. As shown in Figure 1, the 1‐year survival of the cohort was less than 50%, the 5‐year survival was 11%, and the mean postoperative survival was 24 months (95% CI: 11–38 months). There was no association between the primary tumor type and overall patient survival (*p* = 0.300).

### 3.3 Surgery and Adjuvant Radiotherapy

**FIGURE 2:**
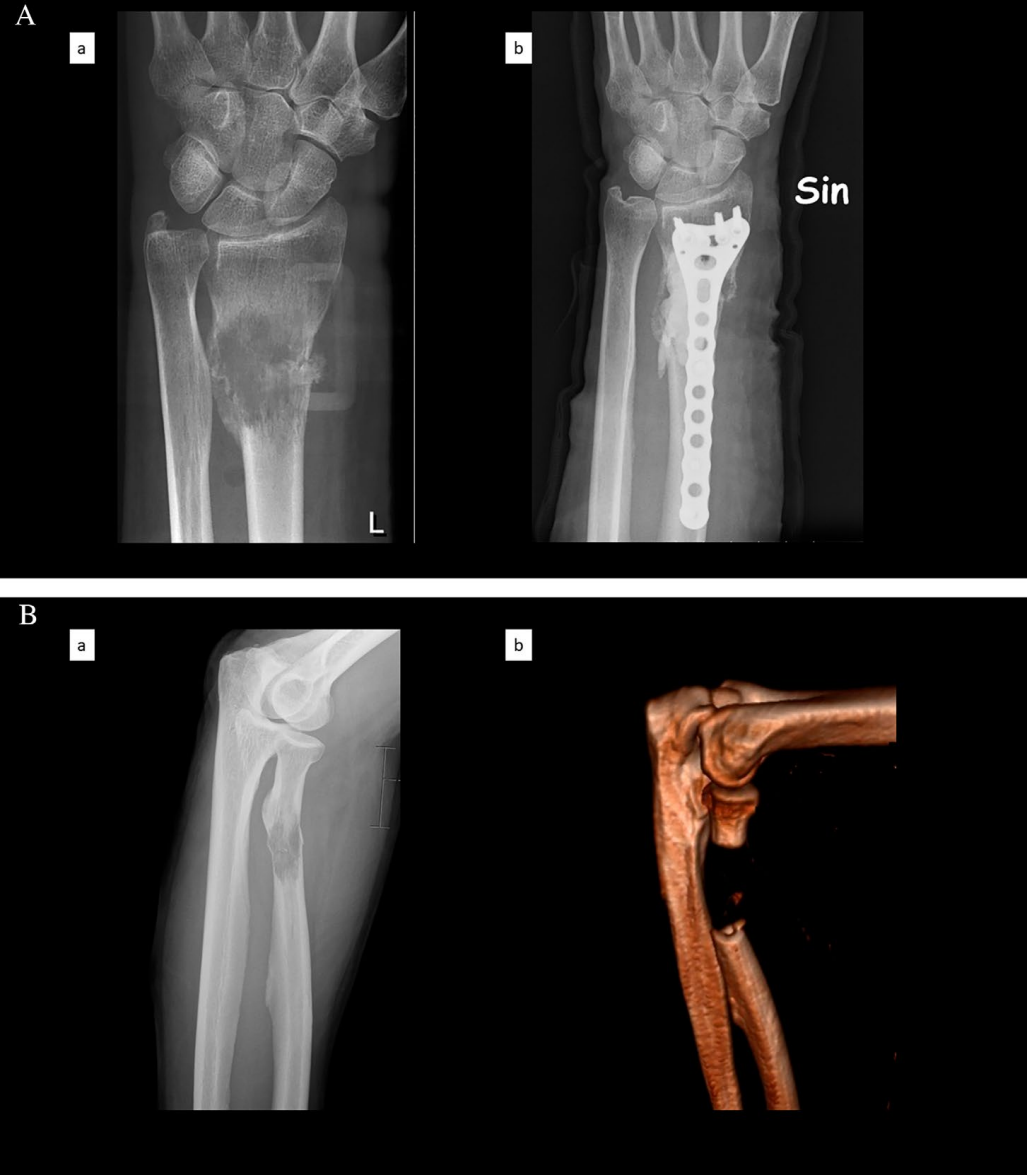
(A) Representative surgical reconstruction of a distal radius metastasis in the metaphysis‐diaphysis transition (a) with curettage, cement, and plate osteosynthesis (b). (B) Representative case of a proximal radius diaphyseal metastasis (a) with segmental excision and no reconstruction (b).

Most of the fractures (*n* = 20) in the present study were treated with osteosynthesis (67%) (Table 2). Of these, 19 fractures were operated on with plates, most of them with cement augmentation (16 fractures), and 1 with intramedullary nails (Figure 2A). The other 10 lesions underwent segmental excisions (five lesions), curettage (three lesions, one with cement augmentation), and amputations (two patients) (Figure 2B). Adjuvant radiotherapy was used in 13 cases (missing information in 3) in doses between 8 and 36 Gy.

**TABLE 2:** Treatment data. Data presented as the frequency of occurred events and percentages (%).

Treatment data		Number (%)
Surgical method	Plate osteosynthesis	19 (63)
	Segmental resection	5 (17)
	Curettage	3 (10)
	Amputation	2 (7)
	Other (pins)	1 (3)
Adjuvant radiotherapy	Yes	13 (43)
	No	14 (47)
	Missing	3 (10)
Status at last follow‐up	Dead	27 (96)
	Alive	1 (4)

### 3.4 Complications and Reoperations

Out of the 30 treated fractures, there were 5 local complications (Table 3). In three of these cases, there were relapses of tumor growth. In one case, the osteosynthesis failed, and the same patient had a local infection. Moreover, there was one case where the cement used to fill the defect dissociated due to tumor progression. Four of these patients were reoperated, and one refrained from further surgery due to modest local symptoms.

**TABLE 3:** Overview of local complications. The table presented each patient with postoperative local complications and reoperation.

	Patient 1	Patient 2	Patient 3	Patient 4	Patient 5
Age at treatment	85	73	67	77	64
Primary malignancy	Merkel‐cell carcinoma	Myeloma	Melanoma	Lung cancer	Renal cancer
Localization	Ulna, proximal	Ulna, diaphysis	Radius, proximal	Ulna, proximal	Ulna, distal
Primary surgery	Plate and cement	Plate	Curettage	Plates and cement	Curettage and cement
Complication type	Tumor growth	Collapse and local infection	Tumor growth	Tumor growth	Collapse
Reoperation	Curettage	Removal implant and new osteosynthesis	New curettage	New osteosynthesis	No reoperation
Time to reoperation	12 months	11 months	6 months	18 months	—

**FIGURE 3:**
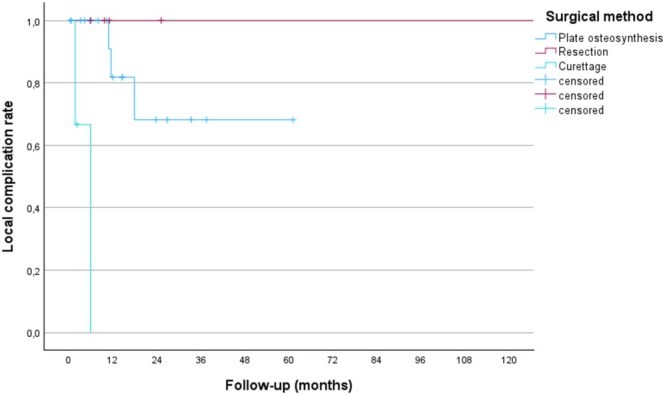
Postoperative local complication rate depending on the mode of surgical reconstruction. Amputations and pin fixation were excluded from the analysis, which was done using the Kaplan–Meier method.

As shown in Figure 3, the primary treatment method was strongly associated with the local failure rate (*p* < 0.001), with segmental resections having excellent outcomes. There was no association between postoperative radiotherapy and adjuvant radiotherapy and postoperative complications or reoperations (data not shown). Only 1 of the 28 patients had a systemic complication, with respiratory insufficiency postoperatively and death in the immediate postoperative period.

### 3.5 Functional Outcome

To further investigate the outcome of surgery, the functional outcome was studied. Of the 16 patients where functional evaluation after surgery was available, 12 (75%) did not report any significant pain, 6 (38%) reported mild or moderate pain, and 1 (6%) had severe pain. Concerning the restoration of mobility, data were available for only 11 patients, of which 10 had minor impairments and 1 significant disability. As data were limited, no comparisons were made between groups, but all three patients with functional follow‐up after segmental excision reported no significant pain and had a near‐normal function.

## 4 Discussion

Given the shortage of reported data regarding pathological forearm fractures, the present study may be a background to aid clinicians and patients in decision‐making, even though it comes with several limitations. Many patients lacked recorded information on postoperative pain, potential pain alleviation, and restored mobility. For functional outcomes, objective measuring tools were not used. Additionally, our data solely comprised sporadic follow‐ups conducted over a wide period, resulting in substantial variations in the follow‐up period among patients. However, the most notable strength of this study is that it is the largest investigation conducted on patients with MBD in the forearm, where only a few case reports have been published previously. Thus, the present cohort provides valuable information for the orthopedic surgeon who will be treating such a lesion, as well as for the patient undergoing surgery.

The current cohort showed a median postoperative survival of 11 months, which is considerably higher than the median survival of 6 months recorded in all the patients operated on for pathological fractures in our Registry. Expected survival is largely dependent on the primary cancer [10, 11] an effect that was not observed in our study due to the small sample size. Moreover, it is interesting that hematologic malignancies, which generally have a good prognosis [12], were overrepresented in our cohort. This could be due to the lower arm mainly consisting of cortical bone, which is less frequently engaged by carcinomas but can be involved in multiple myeloma. Another observation was the relatively low general complication rate, which can be explained by the less extensive surgery and quick mobilization as compared to lower extremity and axial skeleton MBD.

The most common mode of surgical reconstruction was plate osteosynthesis, which can be explained by the anatomy of the bones of the forearm, which are long and mainly cortical. Since these bones have a limited bone marrow compartment, they cannot accommodate rigid nails, and only elastic intramedullary nails or pins can be used. However, they are not the surgical choice of option since pathological fractures generally require stable fixation because they are not expected to heal with bony callus. Interestingly, the forearm contains parts of the skeleton that are dispensable, such as the proximal part of the radius, the distal part of the ulna, as well as limited diaphyseal parts of both bones. In these cases, simple resection may be the best option, and it was successfully used in approximately one‐sixth of the cases in this series, with excellent functional recovery and no reoperations.

The postoperative complication rate was 23%, which is generally in line with previous studies, showing a range of complications from 6% to 35% in MBD patients [6, 7, 8, 10, 13, 14]. The observed local complications were predominantly attributed to tumor relapse. This may be due to the lower arm generally being well‐vascularized and less prone to infection. This is reflected in the rate of local infections in our cohort (3%) being lower than in other studies [8, 10, 13, 14, 15]. Another factor may be the mode of reconstruction in this area, where infection‐prone implants such as prostheses are not used, and many patients were operated without the use of any implant. In the present cohort, no association was shown between adjuvant radiotherapy and a lower frequency of complications or reoperations, due to the lack of power of this study. Despite local complications, the functional outcome was very satisfactory among the patients with available data; pain was absent or minor in the vast majority of the patients, and most had almost completely restored mobility. Segmental resections were associated with very good outcomes, as proof of the principle that many parts of the forearm bones are dispensable and reconstruction is redundant. However, the reoperation rate of 20% was not negligible, but in parity with other studies reporting on lower extremity MBD [16, 17, 18, 19]. The use of minimally invasive techniques has recently been described, and its efficacy as compared to conventional surgery described in this article remains to be proven [20].

## 5 Conclusion

In conclusion, our study is the first to provide evidence regarding the outcome after surgical treatment of pathologic forearm fractures, which aids decision‐making in this challenging context. Patients are generally expected to have a good prognosis, and surgery is well tolerated and provides a good functional outcome. When possible, resection without reconstruction should be preferred.

## Author Contributions

**Jennifer Sebghati:** data curation (equal), formal analysis (equal), visualization (lead), writing – original draft (lead), writing – review and editing (equal). **Panagiotis Tsagkozis:** conceptualization (lead), data curation (equal), formal analysis (equal), investigation (lead), methodology (lead), project administration (lead), resources (lead), supervision (lead), writing – original draft (supporting), writing – review and editing (lead).

## Ethics Statement

This study was approved by the Regional Ethics Committee (2012/272‐31 and 2019‐06189).

## Conflicts of Interest

The authors declare no conflicts of interest.

## Data Availability

Primary data available upon reasonable request.
